# A Novel C1-Esterase Inhibitor Oxygenator Coating Prevents FXII Activation in Human Blood

**DOI:** 10.3390/biom10071042

**Published:** 2020-07-13

**Authors:** Katharina Gerling, Sabrina Ölschläger, Meltem Avci-Adali, Bernd Neumann, Ernst Schweizer, Christian Schlensak, Hans-Peter Wendel, Sandra Stoppelkamp

**Affiliations:** 1 University Hospital Tuebingen, Clinic for Thoracic and Cardiovascular Surgery, Calwerstr, 7/1, 72076 Tuebingen, Germany; Katharina.Gerling@uni-tuebingen.de (K.G.); Sabrina.Oelschlaeger@med.uni-tuebingen.de (S.Ö.); meltem.avci-adali@uni-tuebingen.de (M.A.-A.); Bernd.Neumann@klinikum.uni-tuebingen.de (B.N.); Christian.Schlensak@med.uni-tuebingen.de (C.S.); Hans-Peter.Wendel@med.uni-tuebingen.de (H.-P.W.); 2 University Hospital Tuebingen, Section Medical Materials and Technology, Osianderstr, 2-8, 72076 Tuebingen, Germany; Ernst.Schweizer@med.uni-tuebingen.de

**Keywords:** ECMO, hollow-fiber membranes, C1-esterase inhibitor, anti-thrombogenic, novel coating, inflammation, hemocompatibility

## Abstract

The limited hemocompatibility of currently used oxygenator membranes prevents long-term use of artificial lungs in patients with lung failure. To improve hemocompatibility, we developed a novel covalent C1-esterase inhibitor (C1-INH) coating. Besides complement inhibition, C1-INH also prevents FXII activation, a very early event of contact phase activation at the crossroads of coagulation and inflammation. Covalently coated heparin, as the current anticoagulation gold standard, served as control. Additionally, a combination of both coatings (C1-INH/heparin) was established. The coatings were tested for their hemocompatibility by dynamic incubation with freshly drawn human whole blood. The analysis of various blood and plasma parameters revealed that C1-INH-containing coatings were able to markedly reduce FXIIa activity compared to heparin coating. Combined C1-INH/heparin coatings yielded similarly low levels of thrombin-antithrombin III complex formation as heparin coating. In particular, adhesion of monocytes and platelets as well as the diminished formation of fibrin networks were observed for combined coatings. We could show for the first time that a covalent coating with complement inhibitor C1-INH was able to ameliorate hemocompatibility. Thus, the early inhibition of the coagulation cascade is likely to have far-reaching consequences for the other cross-reacting plasma protein pathways.

## 1. Introduction

Critically ill patients with chronic lung disease are dependent on long-term lung replacement methods. As the demand for donor lungs exceeds the availability [1], an alternative long-term oxygenation system is needed urgently. Currently, extra-corporeal membrane oxygenation (ECMO) is the only treatment option that can be used for a maximum of a few weeks at best [2].

The ECMO circuit consists of a series of components whose key element is the gas exchange membrane. It ensures the exchange of O_2_ and CO_2_. Oxygen-deficient blood is drained from the body to flow along the gas exchange membranes. There, oxygen streams through the inner lumen of the membrane fibers and diffuses through the membrane into the blood, while carbon dioxide is removed [3]. Various membranes can be used for gas exchange [4]. While polypropylene (PP) has good gas permeability properties, plasma-leakage remains a major limitation for long-term use [5,6]. To circumvent the plasma-leakage complications, polymethylpentene (PMP) with its good plasma tightness is most often used for ECMO. In addition to the plasma tightness, PMP is an efficient and low-resistance artificial gas exchange membrane. Further components of the ECMO are the blood reservoir, mechanical blood pump, heat exchanger, and circulatory tube system [3,7].

Despite the benefits of supporting patient oxygenation, using an ECMO/extra-corporeal life support (ECLS) system is also associated with several risks for patients. The most common complications are the occurrence of thromboembolic complications due to the extra-corporeal circuit or bleeding at the cannulation site caused by the required systemic anticoagulation. The large membrane surface is essential for an adequate oxygen supply but can lead to various complications such as acute respiratory distress syndrome (ARDS), systemic inflammatory response syndrome (SIRS), or multi-organ dysfunction syndrome (MODS) [8]. The reason for this is the complex interaction of several blood activation pathways upon contact with foreign surfaces. These include interactions between mechanical and chemical cell activation, dysfunction of the immune regulation and activation of the coagulation cascade. The reaction of the diverse blood components depends on the chemical and physical properties of the materials used (i.e., their hemocompatibility), interactions of the foreign surface with plasma proteins and surface coatings [9,10,11,12,13].

**Figure 1:**
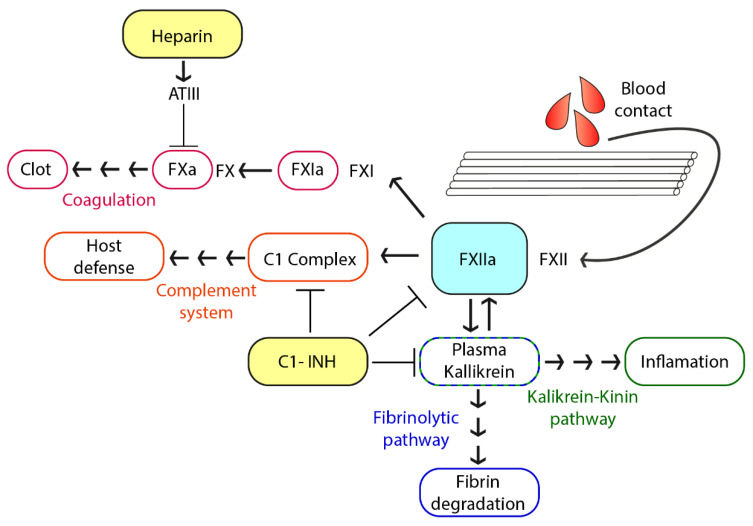
Schematic representation of the contact activation system. The contact of the blood with the artificial surface (polymethylpentene membrane) leads to the activation of factor (F) XII to FXIIa. FXIIa can then activate four different downstream pathways: (i) the coagulation pathway by cleaving the zymogen FXI to FXIa, eventually leading to thrombin activation and fibrin formation, (ii) the complement system by activation of the C1qrs-complex subunits C1r and C1s, (iii) the fibrinolytic pathway by plasma kallikrein activating plasmin to eventually degrade fibrin, (iv) the inflammatory kallikrein-kinin signaling pathway by plasma kallikrein cleaving high-molecular-weight kininogen liberating the pro-inflammatory molecule bradykinin [15]. Heparin and C1-INH are both able to inhibit one or more of these pathways. While heparin only inhibits coagulation, C1-INH positively affects coagulation, the complement system, fibrin degradation, and inflammation [16,24].

The main goal of optimizing ECMO membranes is to develop a non-activating surface [14]. Therefore, activation of coagulation and inflammation can be effectively prevented. The key reaction of coagulation is the thrombin formation that eventually leads to insoluble fibrin clots [15] (see Figure 1). Thrombin generation from prothrombin is mediated by activated factor X (FXa). This key step is inhibited by endogenous serine-protease inhibitor antithrombin III (AT) that targets factor (F) X. Heparin greatly increases the inhibitory function of AT [16]. Thus, heparin-coated oxygenators and tubings as well as systemic heparin are used to prevent activation of coagulation and clot formation. Inflammation on the other hand can be mediated either by anaphylatoxins C3a and C5a generated by the complement pathway or by activation of the kallikrein-kinin pathway [17,18]. None of these are targeted by heparin. On top of this, the crosstalk of the diverse pathways of plasma proteins complicate interventions even further. The contact phase system is activated by autoactivation of FXII on neutral or charged foreign surfaces [19] or by polyphosphates released from activated platelets [20,21]. The following activation of kallikrein leads to the amplification of FXII activation. This in turn activates FXI and forms the link to the coagulation pathway by subsequently activating factors IX and X. Kallikrein on the other hand can digest the high molecular-weight kininogen to liberate the small pro-inflammatory molecule bradykinin [22]. Moreover, FXII and subsequent kallikrein activation have also been reported to occur by misfolded proteins [22,23] suggesting a role of FXII in the induction of inflammation in misfolded protein diseases.

Preventing early reactions, such as the activation of the plasma contact system [25,26], therefore plays an important part here. These pathways are well-known in vitro, but especially the role of FXII in vivo is not fully elucidated yet [19,23]. Diverse coatings in combination with systemic anticoagulation are used to reduce these reactions. The currently used coatings can be divided into non-heparin-based biopassive coatings and heparin-based coatings [14]. For the long-term ECMO mostly heparin-based coatings are used, e.g., Carmeda^®^ BioActive Surface, Bioline coating. A combination of covalent heparin coating and systemic anticoagulation is of clinical benefit as lower doses of systemically applied heparin reduce blood transfusion [27,28].

The anticoagulant effect of heparin can be achieved via a systemic administration or by functionalization of artificial surfaces [27,29]. Covalent coating of tubings and membranes with heparin is especially suited for longer-term use [30]. The mode of action of heparin on the surface is considered either as amplifying the action of AT [31] or as modifying the surface properties towards a more hemocompatible layer [29]. According to Weber and colleagues [29], the effect of heparin coatings on hemocompatibility is probably at least partly due to the adsorption of proteins on the coating, which gives an overall anticoagulant feature. The C1-esterase inhibitor (C1-INH) was one of the proteins adsorbed quite early after blood contact. Since covalent surface modification is a very convenient strategy to achieve a longer-lasting effect, we have established a novel C1-INH covalent coating. C1-INH is an acute-phase protein belonging to the serpin superfamily of serine-protease inhibitors. The primary function of serpins includes the inhibition of proteases. They are also involved in biological interactions with microorganisms and endothelial cells [24,32]. Under physiological conditions, C1-INH can have a positive effect on the complement system, the contact phase system, the kallikrein-kinin system, the fibrinolysis, and the coagulation pathways [33]. In particular, inhibitory effects exerted on FXII and kallikrein make C1-INH a promising candidate for improving ECMO membrane hemocompatibility.

Thus, by coating ECMO membranes with C1-INH, the blood activation and inflammation processes might be reduced. To the best of our knowledge, we are the first group to show the development of a novel covalent C1-INH coating for blood-contacting surfaces. Although the coating is primarily developed for oxygenators to increase hemocompatibility, its design also allows the coating of other surfaces. Another special feature of the coating is that other proteins with a positive impact on hemocompatibility can be covalently bound using the same principle. Thus, to combine the advantages of the current gold standard heparin and the novel C1-INH coating, an additional coating containing both components was also developed. Here, we compared the effect of the covalently coated C1-INH in addition to covalently attached heparin on the hemocompatibility of oxygenator membranes.

## 2. Materials and Methods

### 2.1. Hollow-Fiber Oxygenator Membranes

Polymethylpentene (PMP, OXYPLUS^®^,) hollow-fiber gas exchange membranes with an outer fiber diameter of 380 ± 30 µm and a wall thickness of 90 ± 10 µm, resulting in an inner diameter of 200 ± 50 µm were bought from 3M Membrana (Wuppertal, Germany). PMP membranes with an area of 1 cm^2^ were coated and tested for their hemocompatibility.

### 2.2. Coating of the Membranes

**Figure 2:**
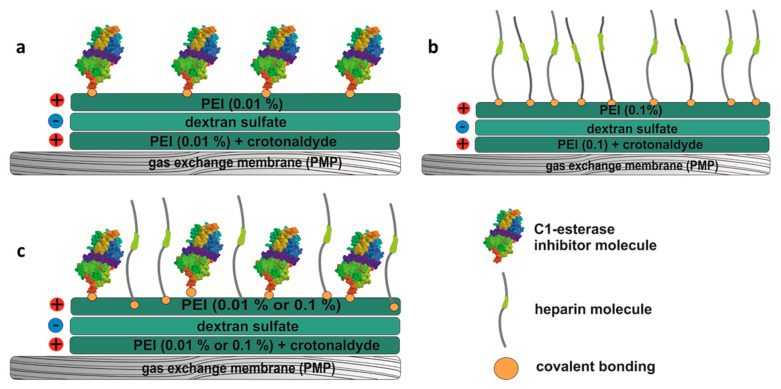
Schematic representation of the generated coatings. (**a**) C1-INH coating, (**b**) heparin coating, (**c**) the combined coating of C1-INH and heparin.

Three different covalent coatings were applied: C1-INH, heparin, and a combination of C1-INH and heparin. A reactive layer of amino groups was generated for all coatings using layer-by-layer technology, originally reported by Decher and Hong on polyelectrolyte multilayers in 1991 [34]. Briefly, the surface was pretreated using 5% ammonium persulfate (55 °C) (Sigma–Aldrich, Darmstadt, Germany). This was followed by alternating layers of the cationic polymer polyethyleneimine (PEI) (0.01% or 0.1%, room temperature (RT)) (Sigma–Aldrich, Darmstadt, Germany) and the anionic polysaccharide dextran sulfate (0.01%, 55 °C) (Sigma–Aldrich, Darmstadt, Germany) in borate buffer (50 mmol/L, pH 8.5) (Thermo Scientific, Waltham, MA, USA). The first layer of PEI was stabilized with 4 mmol/L crotonaldehyde (Sigma–Aldrich, Darmstadt, Germany). After each reaction step, the membranes were washed thoroughly to prevent neutralization of charged molecules. The reaction time for each step was 25 min. The covalent bonding of C1-INH is based on the formation of amidine bonds (see schematic representation in Figure 2a). These were generated by dimethyl suberimidate (DMS) (Thermo Scientific, Waltham, MA, USA) a crosslinker containing two amine-reactive imidoester groups. PMP membranes were placed in a mixture of 3 IU/mL C1-INH (Berinert^®^, CSL Behring, PA, USA) and 3.33 mg/mL DMS dissolved in 0.2 mol/L triethanolamine (Sigma–Aldrich, Darmstadt, Germany) buffer (pH 8.0). After 1 h at RT, the reaction was stopped by incubation with 50 mmol/L Tris buffer (Sigma–Aldrich, Darmstadt, Germany) (pH 8.0) for 30 min. Heparin (Fragmin 2500 IE, Pfizer, New York, NY, USA) (1.6 IU/mL) was covalently bound (Figure 2b) by reductive amination. Here, 0.05% sodium cyanoborohydride (Sigma–Aldrich, Darmstadt, Germany) served as the reducing agent. The reduction reaction was performed at 55 °C in a sterile 9% NaCl solution (VWR Chemicals, Darmstadt, Germany) for 2 h. For the combined coating of C1-INH and heparin (Figure 2c), C1-INH was first covalently bound as described above, followed by the heparin-binding step. After coating, all membranes were washed with isotonic saline solution (0.9%, Fresenius Kabi, Bad Homburg, Germany) and sterile water for injection (Ampuwa; Fresenius Kabi, Bad Homburg, Germany) before drying in a desiccator.

### 2.3. Detection of Bound C1-INH and Heparin

For the detection of surface-bound C1-INH on the hollow-fiber membranes, the TECHNOCHROM^®^C1-INH kit (Haemochrom Diagnostica, Germany) was used. The membranes (1 cm^2^) with immobilized C1-INH were placed in a 24-well plate and filled with sample buffer from the kit to equalize the volume to that of the standard dilutions of C1-INH. The subsequent steps were performed according to the manufacturer’s instructions. Biologically active heparin concentrations were determined by the Chromogenix Coamatic Heparin kit (Haemochrom Diagnostica, Germany) according to the manufacturer′s instructions.

### 2.4. Blood Sampling

Blood was obtained from five voluntary healthy adults by venipuncture (Safety-Multifly^®^ 20 Gx3/4 TW needle; Sarstedt, Nümbrecht, Germany) after giving their informed consent. To enable conclusive in vitro blood experiments, relatively low anticoagulation with 1 IU/mL sodium heparin 25,000 (Ratiopharm, Ulm, Germany) was used. To guarantee the quality of the blood, strict exclusion criteria were applied to blood donors: e.g., smoking and medication were prohibited. In particular, the intake of drugs inhibiting coagulation and inflammation in the last two weeks before blood donation was not allowed. The blood collection procedures described and the use of blood in the experimental settings were approved by the Research and Ethics Department of the University of Tuebingen (project approval number 287/2020BO2).

### 2.5. Blood Incubation

To demonstrate the anti-inflammatory and anti-thrombogenic effects of the coatings, the differently coated membranes and an untreated membrane (each 1 cm^2^) were dynamically incubated with 9 mL freshly drawn heparinized human whole blood in polypropylene tubes (Becton Dickinson (BD) Biosciences, Heidelberg, Germany) at 37 °C for 90 min. Heparinized blood incubated without sample material served as control. Basal activity of each measured marker was obtained from blood that was transferred into the corresponding anti-coagulated monovettes immediately after collection. Changes to the basal levels were determined in dynamically incubated blood samples after 90 min. Blood cell counts (platelets, erythrocytes, and lymphocytes) were measured directly with an automated cell counter (ABX Micros 60, Horiba Medical, Kyoto, Japan). All other activation markers were evaluated from plasma that was shock-frozen and stored at −20 °C or −80 °C, depending on the manufacturer’s instructions. For the FXIIa activity test, the heparin was neutralized with protamine (ME 1000 I.E/mL, MEDA, Solna, Sweden). Anti-coagulating reagents were: Ethylenediaminetetraacetic acid (EDTA; Sarstedt, Nümbrecht, Germany) for blood cell count measurement and complement enzyme-linked immunosorbent assay (ELISA), CTAD (a mixture of citrate, theophylline, adenosine, and dipyridamole; BD Biosciences, Heidelberg, Germany) for platelet activation ELISA, and citrate (Sarstedt, Nümbrecht, Germany) for FXIIa activation test, thrombin-antithrombin III complex ELISA, and leukocyte activation ELISA.

### 2.6. Soluble Activation Markers

Changes in the activation of the complement system, contact phase system, blood coagulation, platelets, and monocyte activation were detected by ELISA for the corresponding activation markers. All ELISAs were performed according to the manufacturer′s instructions. C3a fragment (C3a MicroVue™, Quidel, Osteomedical GmbH, Sissach, Switzerland) was determined from EDTA plasma, while thrombin-antithrombin III complex (TAT) (Enzygnost^®^ TAT micro, Siemens Healthcare, Erlangen, Germany) and polymorphonuclear (PMN) elastase (PMN Elastase ELISA, Demeditec Diagnostics, Kiel, Germany) were measured in citrated plasma. Activation of platelets was quantified by β-thromboglobulin (β-TG) release (Asserachrom^®^ β-TG, Diagnostica Stago, Parsippany, NJ, USA) in CTAD-anticoagulated plasma. Blood coagulation was measured with an assay for Factor XIIa-like activity (Unitest Factor XIIa-like activity, Haemochrom Diagnostica, Germany).

### 2.7. Scanning Electron Microscopy

After blood incubation, the membranes were rinsed thoroughly with 0.9% saline solution (Fresenius Kabi, Bad Homburg, Germany) until all excess and non-adsorbed blood components were removed. The rinsed PMP membranes were fixed for one day in 2.5% glutaraldehyde (Sigma–Aldrich, Darmstadt, Germany) in phosphate-buffered saline (PBS; Thermo Scientific, Waltham, USA). Dehydration was performed by an ascending ethanol series before mounting and critically point drying. Dried samples were finally sputtered with gold-palladium particles (Baltec SCD 050, Bal-Tec AG, Balzers, Liechtenstein) and imaged by a scanning electron microscope (LEO 1430, Zeiss, Oberkochen, Germany). In each case, 100-fold, 500-fold, and 1000-fold magnifications were taken.

### 2.8. Fluorescence Microscopy

Surface cell adhesion was determined by 4′,6-Diamidine-2′-phenylindole (DAPI) cell staining. The surface was washed thoroughly with 0.9% saline solution and the adsorbed cells fixed in formaldehyde (4%; Fischar, Saarbrücken, Germany) for at least one hour. To stain the cells, the samples were incubated in 0.2 µg/mL DAPI (Sigma–Aldrich, Darmstadt, Germany) in PBS for 5 min. The adherent cells were visualized by fluorescence microscopy (Optiphot-2 Nikon) equipped with a remote control digital single-lens reflex camera (Nikon 550 D, Nikon, Japan). Quantification was performed via the program “ImageJ” from 20 independent images per parameter at 10x objective magnification. For quantification, the background was subtracted by setting the rolling ball radius to 50 pixels, setting a threshold, and counting the colored particles with a size (pixels^2^) of 250 to infinity.

### 2.9. Statistical Analyses

Experiments were repeated from five independent samples (donors). Obtained results were first tested for normal distribution by the Shapiro–Wilk normality test. All data sets showing normal distribution were analyzed with a one-way analysis of variance (ANOVA) with Bonferroni’s multiple comparison post hoc test to ascertain differences between the groups (hemocompatibility parameters). The Kruskal–Wallis test with Dunn′s multiple comparison test was used for non-normal distributed datasets. Statistical significance was defined as *p* < 0.05. All analyses were performed using the statistical software package GraphPad Prism version 6.01 (GraphPad Software Inc., La Jolla, CA, USA). Non-marked bars are considered not significant to each other.

## 3. Results

### 3.1. Quantification of Immobilized C1-INH and Heparin on PMP Membranes

**Figure 3:**
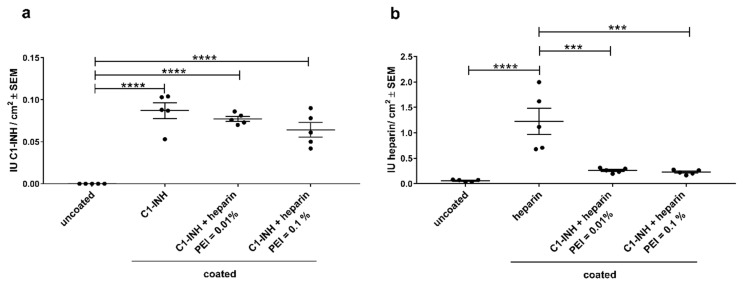
Detection of bioactive (**a**) C1-INH or (**b**) heparin amounts on PMP membranes. Significantly increased amounts of C1-INH were detected compared to the uncoated samples. However, only heparin-coating resulted in significantly increased immobilization of heparin compared to untreated samples. The results are shown as mean ± scanning electron microscopy (SEM). Statistical analysis was performed using one-way ANOVA with Bonferroni’s multiple comparison post hoc test. *n* = 5; **** *p* < 0.0001, *** *p* < 0.001.

Covalent attachment of the three different surface modifications was quantified by modified C1-INH and FXa assays. Similar amounts of covalently coated bioactive C1-INH could be detected when PMP membranes were coated with C1-INH alone (0.087 ± 0.021 IU/cm^2^) or in combination with heparin (Figure 3a). Combined C1-INH/heparin coatings were tested with two different PEI concentrations (0.1% and 0.01% PEI). The incorporation of PEI did not significantly influence the conjugated C1-INH amount on PMP membrane surface, but the lower PEI concentration led to more homogenous amounts of coated C1-INH. In contrast, in combined coatings, heparin concentration was significantly lower than the coatings with only heparin (Figure 3b). Heparin amounts dropped from 1.143 ± 0.688 IU/cm^2^ for only heparin coatings to 0.226 ± 0.046 IU/cm^2^ (PEI = 0.1%) and 0.261 ± 0.047 IU/cm^2^ (PEI = 0.01%) for combined coatings. This effect was likely due to the sequential coating of C1-INH before heparin in combined coatings. Overall, we could successfully immobilize bioactive C1-INH and bioactive heparin on PMP membrane surfaces.

### 3.2. Analysis of Hemocompatibility

An ideal coating should confer near-natural properties to oxygenator membranes, minimizing coagulation and inflammatory activation of blood. To evaluate the impact of the coated membranes on the diverse blood components, e.g., coagulation and complement system, and activation of blood cells, differently coated PMP membranes were incubated dynamically with heparinized, fresh human whole blood. After incubation, the blood cell counts, and various hematological markers were determined and compared between coatings and to an uncoated membrane. Variations in blood cell count indicate cell loss due to adhesion or activation of platelets, adhesion of leukocytes, or hemolysis of erythrocytes.

**Figure 4:**
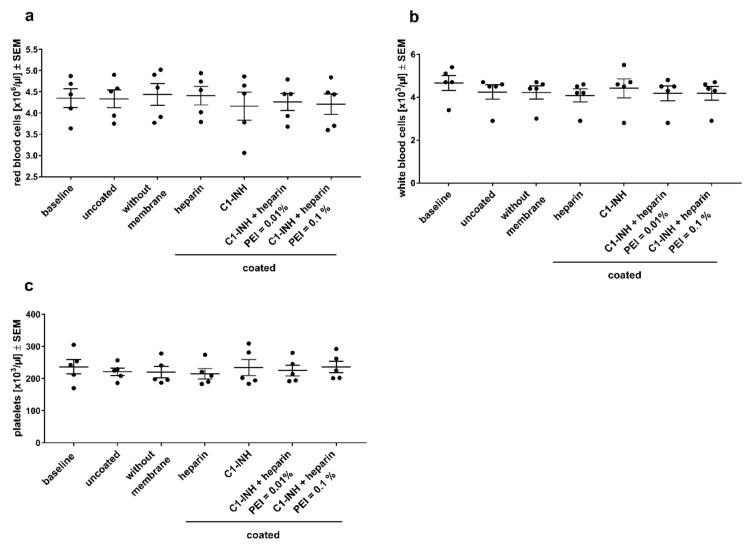
Comparative analyses of the blood cell number before (baseline) and after dynamic incubation revealed no significant loss of leukocytes and platelets after incubation with either coated or uncoated membranes. Shown are mean numbers of red blood cells (RBCs) (**a**), white blood cells (WBCs) (**b**), and platelets (**c**) per microliter heparinized whole blood ± SEM. Statistical analysis was performed using one-way ANOVA with Bonferroni’s multiple comparison post hoc test (**a**,**c**), or Kruskal–Wallis test with Dunn′s multiple comparison post hoc test (**b**). *n* = 5.

The number of erythrocytes was not significantly different before (baseline) and after dynamic incubation (Figure 4a), indicating no destruction of erythrocytes by hemolysis due to material contacts or the incubation procedure. There was no reduction of white blood cell numbers compared to the controls without a membrane (baseline = before incubation; control = after incubation) (Figure 4b). Similarly, no reduction of platelet counts was observed for all coated membranes and the uncoated membrane (Figure 4c). This shows that all membranes, coated and uncoated, do not lead to marked cell loss or hemolysis.

**Figure 5:**
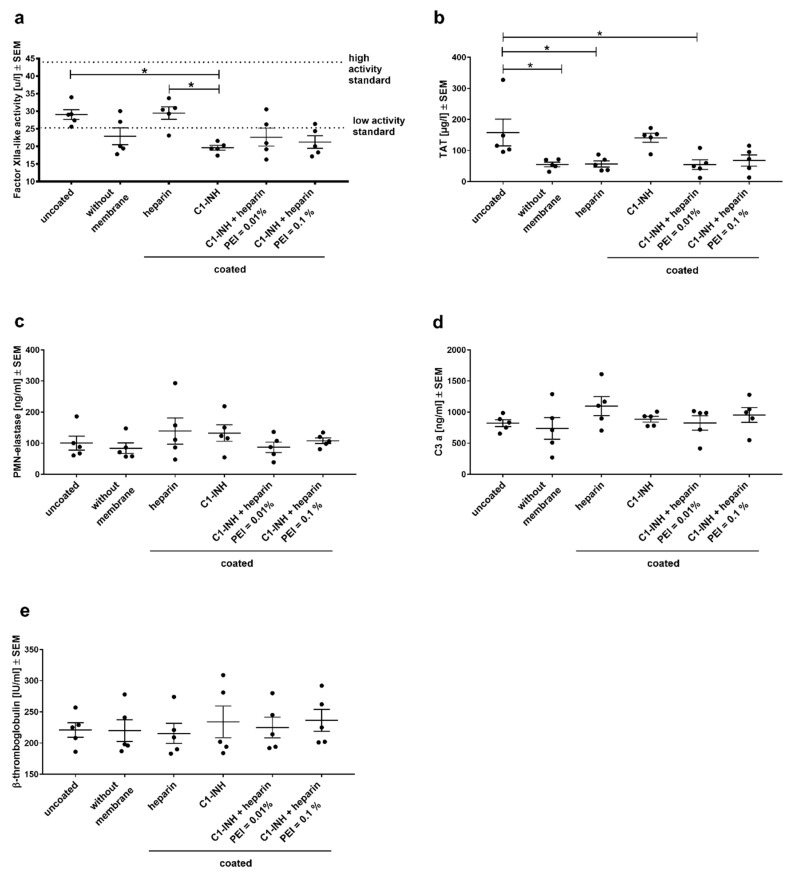
C1-INH coating prevented FXII activation, while heparin coating prevented TAT formation. Hemocompatibility analysis of differently coated PMP membranes: blood markers for the contact phase system (FXIIA-like activity) (**a**), coagulation (TAT) (**b**), activation of neutrophils (PMN-elastase) (**c**), complement system (C3a) (**d**), and platelet activation (β-thromboglobulin) (**e**). Statistical analysis was performed using one-way ANOVA with Bonferroni’s multiple comparison post hoc test. The mean value of each column was compared with the mean value of each other column. *n* = 5; * *p* < 0.05.

Activation of the different cross-reacting blood pathways (contact phase system—FXIIa-like activity; coagulation—TAT; complement system—C3a; platelet activation β–TG; leukocyte activation PMN-elastase) was determined from freshly frozen plasma samples (Figure 5). Comparing blood without a membrane (control) and blood incubated with uncoated membranes indicates the effect of the material PMP itself. A clear effect was observed for contact phase activation (Figure 5a) and coagulation (Figure 5b). The C1-INH coating significantly reduced FXII activation compared to heparin coating and uncoated membranes. The combined coatings of heparin and C1-INH showed a larger variation between donors, thus no significant differences were seen compared to heparin coating or C1-INH coating, but mean FXIIa activity was on the same level as blood without membrane. Significantly reduced TAT complex generation was detected after the incubation of blood with only heparin or C1-INH/heparin (PEI 0.01%) coated membranes compared to uncoated membranes (Figure 5b). Heparin-coated membranes were able to significantly reduce TAT formation back to control levels. The significant reduction of TAT complex back to the same low TAT levels as heparin by the combined coating (PEI 0.01%) shows the effective prevention of thrombin formation by C1-INH/heparin coating. There were no significant differences measured between the coatings regarding neutrophil granulocytes (Figure 5c), complement system (Figure 5d), and platelet activation (Figure 5e).

### 3.3. Membrane Surface Analyses

**Figure 6:**
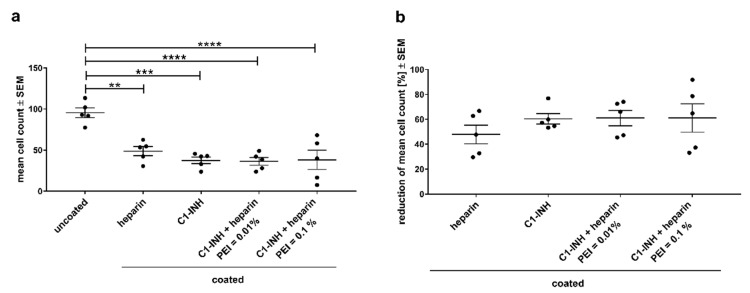
Fluorescence microscopy revealed reduced cell adhesion on single C1-INH and heparin as well as combined coatings. Mean cell counts ± SEM of DAPI-stained membranes show the significant reduction of nucleated cells on the surface of coated membranes (**a**), proportional reduction of mean adhered nucleated cells visualize the effect in direct comparison of the different coatings (**b**). Statistical analysis was performed using one-way ANOVA with Bonferroni’s multiple comparison post hoc test. The mean value of each column was compared with the mean value of each other column. *n* = 5; **** *p* < 0.0001, *** *p* < 0.001, ** *p* < 0.01.

Activation of cells usually precedes cellular adhesion and aggregation. This later event of cell adhesion was investigated using fluorescence microscopy of DAPI-stained membranes and using scanning electron microscopy (SEM). DAPI-staining was supposed to indicate the number of nucleated cells (mainly neutrophils) attached to the membranes after blood contact. Figure 6a shows the mean number of adhered cells of representative fluorescence microscope images. All coatings significantly reduced the cell adhesion compared to the untreated membrane, but cell adhesion did not differ between coatings. The proportional amount of reduction is visualized in the scatter plot in Figure 6b. Interestingly, while heparin reduced cell adhesion significantly (*p* < 0.01), the reduction by C1-INH-containing coatings was highly significant (*p* < 0.001) indicating an additional effect of C1-INH to reduce adhesion of nucleated cells.

**Figure 7:**
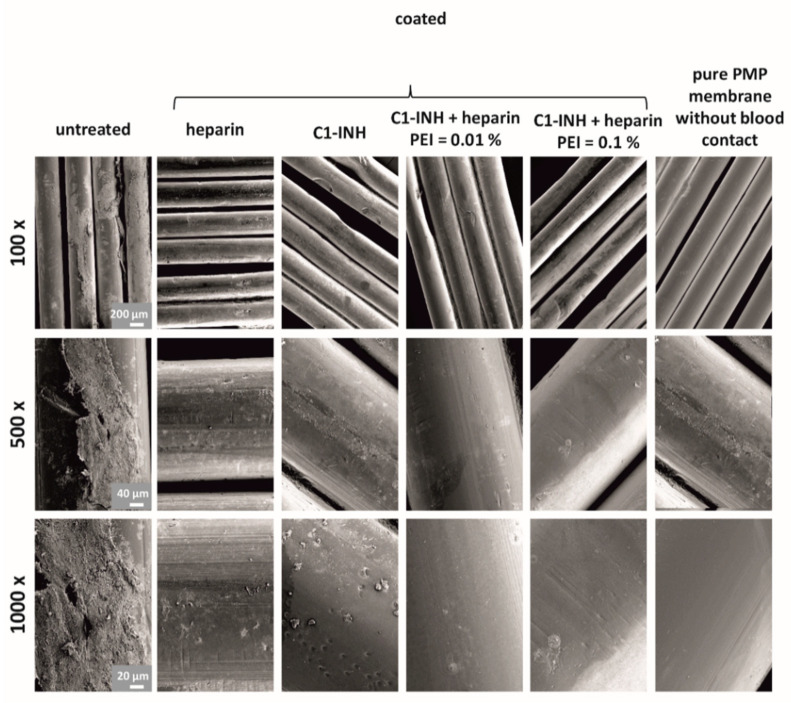
SEM images show an obvious decrease in platelet aggregation and fibrin formation. This effect is particularly noticeable with the combined coatings of heparin and C1-INH. For each type of coating, images were taken with a 100×, 500×, and 1000× magnification. For a visual comparison of the effects, images of an uncoated PMP membrane without blood contact are also shown (last column).

In addition to the adhesion of neutrophils, there is typically a rapid adhesion and aggregation of activated platelets on artificial surfaces, which is associated with fibrin adhesion and clot formation. These events were investigated using SEM. All coatings significantly reduced adhesion and aggregation of activated platelets as well as fibrin adhesion and clot formation. Particularly few deposits can be seen in the combined coatings (Figure 7). The combination of C1-INH and heparin was able to prevent platelet aggregation and fibrin formation almost completely. However, in the untreated membrane, several adhered platelets and large fibrin networks were visible.

## 4. Discussion

This study aimed to improve the hemocompatibility of ECMO membranes by using a novel coating that inhibits early events in the activation of plasma proteins. Our approach was to use the serine-protease inhibitor C1-INH, a plasma protein acting as a complement inhibitor as well as an inhibitor of other signaling pathways such as the contact system, fibrinolysis, and coagulation [33]. The usual protein interactions are reported to change in their bound state [35]. Although this mechanism is not fully described yet, it is assumed that this is dependent on the adsorption-related conformational changes of proteins [29]. Hylton at al. showed that the binding of fibrinogen and serum albumin increases the tendency to thrombus formation [35]. However, the change in the mode of action of bound proteins does not necessarily have to be accompanied by negative effects. Weber et al. showed that at least part of the improved blood compatibility of heparin-coated biomaterials is due to the selective uptake and cleavage of plasma proteins [29]. It was also shown that increased adsorption of C1-INH took place on surfaces coated with heparin. This indicates a positive influence of bound C1-INH on blood compatibility [29].

In the present study, such a positive effect of C1-INH coating was also detected. We could show that coating with C1-INH improves hemocompatibility. This is particularly evident in the pronounced inhibition of Factor XIIa by C1-INH coating. Here, the heparin coating did not show a marked reduction in FXIIa activity. In contrast, the very early event in activation of several blood plasma pathways by FXII was significantly blocked by C1-INH. Particularly coagulation and inflammation are dreaded adverse events in ECMO. An increase in the formation of thrombin-antithrombin III (TAT) complex in plasma is proportional to the activation of FX, since FXa (as catalytic part of the prothrombinase complex) cleaves prothrombin to thrombin that is captured by AT to form the TAT complex [36,37]. As expected, heparin, which is known to enhance the effect of AT, significantly reduced TAT plasma levels. C1-INH coating on the other hand did not show a similar effect, indicating that the possible FX activation for increasing TAT plasma levels was not solely caused by FXII and its downstream actions. Although the initiation of coagulation by medical devices is often reported to start with FXII activation leading to downstream activation of FXI, FIX and FX [38,39], this exact sequence of events seems unlikely here, since FXII was significantly inhibited by C1-INH coating, while TAT levels were not reduced. It is possible that the activation of the coagulation was started independently of FXII by FXI autoactivation. FXI itself was reported to be autoactivated by negatively charged surfaces and the activation was accelerated by thrombin [40]. Thus, a small amount of thrombin formed can autoactivate FXI leading to further downstream events. FXI is also a target of C1-INH [24], therefore either the amount of C1-INH was not sufficient to block both FXII and FXI or there have been other activating events. Looking at the combined coatings of C1-INH and heparin, both showed similar TAT levels as heparin coating alone although the amount of heparin was approx. 3-times lower (compare Figure 2). Thus, the incorporation of C1-INH into heparin coating indicates an improvement of heparins action on the surface.

Although there was no obvious difference in activation of neutrophils (PMN-elastase), complement activation (C3a), and platelet activation (β-TG) between coatings, there was a significant reduction of leukocyte attachment to the coated membranes (Figure 6). The combined coatings of C1-INH and heparin even showed a highly significant decrease in leukocyte binding. This suggests that the adhesion of cells could be further decreased by the incorporation of C1-INH into heparin coatings. The investigation of fibrin formation and platelet adhesion by SEM reinforces this assumption. Almost no platelet adhesion or fibrin networks were seen on membranes coated with C1-INH and even less in combined coatings (Figure 7). Heparin-coated membranes showed decreased adhesion of platelets and generation of fibrin networks than uncoated membranes, but there were more accumulations seen than on the other coated membranes. This result demonstrates that there must be another underlying effect of C1-INH coating than preventing activation of coagulation. The adhesion of plasma proteins may be one of the underlying events. As reported previously [29], the adsorption of C1-INH was detected on heparinized surfaces. The authors suggested a role of adsorbed C1-INH and other proteins for improved hemocompatibility of heparinized surfaces. By directly coating C1-INH to the surface of PMP membrane, the adsorption of plasma proteins may have been steered towards a hemocompatible non-activating surface. Moreover, FXII can be activated by misfolded proteins [41]. Inhibition of FXII activation by C1-INH could therefore also reduce the negative effect of proteins that adsorb to the surface and undergo a change in conformation. This activation of FXII would usually promote unwanted inflammation, whereas preventing protein adsorption and misfolding on the surface can reduce the binding of fibrinogen and platelets to the surface [42]. The mechanism of bound C1-INH preventing platelet and leukocyte adhesion is not clear but should be investigated further. In particular, the role of protein adsorption to the surface and direct interaction of diverse coagulation factors with C1-INH are of particular interest to understand the observed reduction in cell adhesion. Irrespective of the mechanism, the direct advantage of C1-INH coating for a group of patients reacting to heparin is directly obvious. In some cases, patients can develop heparin-induced thrombocytopenia (HIT) when exposed to heparin. Although type 1 of this disease is non-immune mediated and the drop in platelet counts stabilizes during continuous heparin treatment [43], type 2 HIT is an immune reaction with life-threatening thromboembolic complications [44]. IgG antibodies are produced within 5 days and form a complex with heparin and platelet factor 4 leading to platelet aggregation and thrombosis [45]. The occurrence of such IgG is common, whereas HIT type 2 is relatively rare [46]. Nevertheless, substituting heparin coating with C1-INH could prevent HIT and make ECMO safer for such patients. In the case of HIT, the formed antibodies can lead to the activation of the classical complement pathway leading to inflammation [47]. Thus, the presence of C1-INH in combined C1-INH/heparin coating could prevent antibody- and C1-induced complement activation if HIT would occur.

## 5. Conclusions

Our novel covalent C1-INH coating demonstrated improved hemocompatibility by inhibition of FXII activation. The reduced adhesion of leukocytes and platelets was obvious for all types of coatings; however, C1-INH/heparin coatings showed the lowest adhesion of cells. As the membrane sizes in this in vitro study is relatively small compared to the full-sized oxygenator, we expect an even more distinct effect in large-scale models. The combined C1-INH/heparin coatings showed the most promising results in preventing platelet adhesion and fibrin networks. Therefore, these combinations will be extended in different ratios in the next study to reveal the optimal ratio of coated heparin to C1-INH before larger-scale models are investigated. Furthermore, the mechanism behind C1-INH coating leading to the observed events will be analyzed in more detail by biochemical methods.
